# Using a novel endoprosthetic device to modulate mechanical strain in gyroid scaffolds in large bone defects

**DOI:** 10.3389/fbioe.2026.1948003

**Published:** 2026-10-27

**Authors:** Shashank Kanakamedala, Paul Heyliger, Tyler Gerry, Daniel Watling, David Prawel

**Affiliations:** 1 Department of Mechanical Engineering, Colorado State University, Fort Collins, CO, United States; 2 Department of Civil and Environmental Engineering, Colorado State University, Fort Collins, CO, United States; 3 School of Biomedical and Chemical Engineering, Colorado State University, Fort Collins, CO, United States

**Keywords:** bone biomechanics, critical bone defect, endoprosthetic, gyroid scaffold, polycaprolactone sleeve, strain gauge

## Abstract

This study investigates the strain behavior of highly porous gyroid scaffolds composed of pure deproteinized ovine bone mineral (OBM) and evaluates the role of a novel endoprosthetic “sleeve“ in modulating mechanical response in ovine cadaveric bone with a critical size defect stabilized by a Locking Compression Plate (LCP) under physiological loading. Scaffold-sleeve assemblies were constructed by inserting scaffolds into sleeves which were implanted into critical metatarsal segmental defects. The scaffold-sleeve constructs were stabilized with locking compression plates (LCP) and subjected to cyclic compressive loading (0–150 N). Measurements were made on surrogate PLA scaffolds and then translated to bioceramic scaffolds. Strains were measured using three-element 0°/±45° rosette gauges mounted on scaffolds with and without the sleeve. Results indicated that scaffold strains ranged between 200 and 300 µε, comparable to strain levels in native bone without defects. Notably, the presence of the endoprosthetic sleeve significantly reduced transverse strain by 8.2% to approximately 0.03%. These findings show that the inclusion of an endoprosthetic sleeve can effectively modulate local strain environments of highly porous scaffolds in large bone defects.

## 1 Introduction

Traumatic bone injury represents a significant clinical and economic burden on global healthcare systems, with incidence rates escalating due to aging populations, increased mobility, and higher rates of osteoporotic fractures. In particular, critical-sized bone defects; defined as osseous voids of a magnitude that precludes spontaneous regeneration, account for a substantial proportion of these cases (Schemitsch, 2017). These defects are most commonly associated with high-energy trauma, tumor resection, infection, or congenital anomalies, and their management remains a formidable orthopedic challenge due to impaired vascularization, mechanical instability, and compromised regenerative potential.

To date, bone grafting remains the clinical mainstay for reconstructive intervention in large defects. Autografts, harvested from the patient’s own body, are considered the gold standard owing to their osteoconductive, osteoinductive, and osteogenic properties. However, their application is hindered (Younger and Chapman, 2026; Schmidt, 2021) by donor site morbidity, prolonged operative time, and limited tissue availability, especially in treatment of large bone defects. Allografts, although more readily available, carry risks (Campana et al., 2014; Ng, 2012) of immune rejection, disease transmission, and reduced integration. These limitations underscore the urgent need for advanced remediation strategies that can replicate the complex mechanical (Claes et al., 1998) and biological milieu required for functional bone regeneration.

Tissue-engineered scaffolds have emerged as a promising solution to this problem, offering the ability to tailor mechanical properties, biodegradability, and bioactivity to the needs of the defect site. Among these, calcium phosphate-based materials, particularly apatites, are favored due to their chemical similarity to the mineral phase of bone, supporting osteointegration and cellular attachment. Despite their osteoconductive superiority, apatite scaffolds are characteristically brittle and possess insufficient fracture toughness for load-bearing applications, severely limiting their clinical translation in critically sized reconstructions. The scaffolds are typically implanted using an interference fit in the defect site, relying on direct mechanical contact between host bones, surrounding soft tissue and the scaffolds to restrain them in the defect site. The scaffold receives only minimal lateral support from the surrounding soft tissue, resulting in constant change in position when undergoing dynamic loading (Pilia et al., 2013).

Recent advances in scaffold architecture, particularly the triply periodic minimal surface (TPMS) topologies, have provided new avenues to address these limitations (Lu et al., 2019). TPMS topologies such as the gyroid offer high porosity, interconnected pore networks, and smooth continuous internal surfaces that help minimize stress concentrations. These characteristics facilitate nutrient transport and cell infiltration while improving mechanical stability (Melchels et al., 2010; Castro et al., 2019). However, while TPMS architecture enhances structural efficiency, they do not enable the toughness required to support human-scale loading (CAO, 2022).

To address this, countless reinforcement strategies have been explored, many involving polymeric materials either through composite fabrication or external coating. While these modifications confer improved mechanical integrity, they often compromise the osteogenic responsiveness of the scaffold by attenuating surface bioactivity and hindering cell-material interactions (Yang et al., 2021; Kutrolli et al., 2026; Liang et al., 2024). When polymers and other compositing materials are incorporated into the scaffold to provide mechanical reinforcement, they often occupy pore space and coat surfaces that would otherwise be available for bone cell attachment and remodeling. This trade-off between mechanical robustness and osteogenic potential remains a design dilemma in scaffold development. Beyond immediate mechanical performance, the long-term fate of any implanted polymeric device must align with adequate bone healing to avoid premature loss of mechanical support or prolonged requirement for fixation.

Successful bone regeneration depends not only on osteogenic biomaterials and biological cues but also on the mechanobiological environment within the defect, particularly the local strain experienced by cells and newly forming tissue. Mechanical strain strongly influences the balance between osteogenesis, fibrous tissue formation, and endochondral *versus* intramembranous repair, with moderate, well-controlled deformation generally supporting healing while excessive strain can delay regeneration and impair tissue quality. Maintaining very low strain within the scaffold has been associated with increased intramembranous bone formation (Claes and Heigele, 1999; Pobloth et al., 2018) and promotes osteoblast activity and proliferation, extracellular matrix deposition, and the formation of a stable repair tissue architecture. Strain also plays a critical role in neo-vascularization, as mechanical conditions that support tissue ingrowth and perfusion facilitate endothelial cell migration, vessel maturation, and the delivery of oxygen and nutrients needed for successful bone formation. Conversely, an overly rigid or overly compliant environment can disrupt this balance, limiting both vascular infiltration and new bone deposition. Scaffold stability is also a key mechanobiological factor, particularly in the transition zones, but ideal ranges are unclear in critical defect remediation. Rigid stabilization to reduce inter-segmental mobility will increase the rate and quality of ossification in the transition zones (Marsell and Einhorn, 2011), whereas greater mobility can prolong healing (Epari et al., 2006) by extending the chondral phase and reducing callus strength and stiffness and promoting fibrous tissue formation (Pobloth et al., 2018; Schell et al., 2005). Excessive motion and/or load delays healing (Green et al., 2005).

To accommodate these factors, we propose a novel conceptual framework wherein the polymeric component is physically decoupled from and positioned externally to the scaffold. This strategy aims to provide mechanical support and reinforcement without altering the osteogenic properties of the scaffold, thereby preserving its bone regeneration capacity. Moreover, by reducing the strain experienced by the scaffold, an external polymeric component could enable a mechanobiological environment more conducive to bone growth. The central hypotheses of this study are that the endoprosthetic sleeve can modulate the local strain environment in a highly porous scaffold.

To test this hypothesis, we conducted a study utilizing an ovine critical segmental defect model employing highly porous gyroid scaffolds in conjunction with an externalized polycaprolactone sleeve, with the objective of quantifying the sleeve’s effect on the scaffold transverse surface strain magnitude under physiological compressive loading. This approach aims to advance the translational potential of brittle but highly osteogenic ceramic biomaterials for the treatment of critical sized bone defects.

## 2 Materials and methods

### 2.1 Scaffold fabrication and strain gauge attachment

Gyroid scaffold geometries were developed using the implicit surface parameterization method described by Baumer et. al. (2024), shown in Equation 1, specifically adapting their mathematical framework for generating continuous gyroid unit cells at defined porosity and pore size. Two types of specimens were fabricated for this study. First, 30 mm cubic specimens were printed using PLA filament for material characterization. These cubes were designed with a gyroid topology at approximately 70% porosity and 1 mm pore size, with a wall thickness of 0.413 mm (single road width, equal to nozzle diameter), and were compression tested on a Tinius Olsen H1KS 1kN benchtop mechanical testing machine (Tinius Olsen, Pennsylvania, USA) to determine the Young’s modulus and Poisson’s ratio of the PLA material. The second specimens created were anatomically shaped scaffolds that were printed to replicate the geometry of the ovine metatarsal segment being replaced, maintaining identical gyroid topology, porosity, and printing parameters for use in the cadaveric bone assembly testing. All specimens were fabricated using a Bambu P1S 3D printer (Bambu Lab, China) with the following parameters: layer height 0.2 mm, print speed 40 mm/s, nozzle temperature 210 °C, and bed temperature 60 °C. Finalized geometries were exported as STL files and processed in Ultimaker Cura slicing software (Ultimaker B.V., Utrecht, Netherlands) to generate the G-code toolpaths used for fabrication, in accordance with Equation 1.

$$gyroid\left(x,y,z\right)=\sin\left(y\right)\cos\left(x\right)+\sin\left(z\right)\cos\left(y\right)+\sin\left(x\right)\cos\left(z\right)=0$$ (1)

Scaffolds were fabricated using a melt extrusion additive manufacturing process on the Bambu printer utilizing polylactic acid (PLA) filament. The scaffold geometry was divided into distal and proximal interlocking regions which were connected through crenellated junctions designed to ensure mechanical continuity, intersegmental immobility and ease of assembly. Following printing, the outer facing surface of each scaffold was manually sanded using 150-grit sandpaper to create a uniform, planar surface suitable for strain gauge mounting. Surface dust was removed by wiping the scaffold with isopropyl alcohol to ensure optimal adhesive bonding, which was tested for electrical integrity.

Rosette strain gauges (C4A-29-031WWA-350), shown in Figure 1, from Micro-Measurements (North Carolina, USA) were used to capture multiaxial strain data from the scaffold surface (Cordey and Gautier, 1999; Clark et al., 2020). Each strain gauge was first mounted on a glass slab to maintain flatness and orientation, temporarily secured in position using low-residue alignment tape (PCT-3M, Micro-Measurements, North Carolina, USA). MBond 200 adhesive was employed both as a surface sealant and bonding agent (Komurlu et al., 2016). A thin layer of MBond 200 was applied to a region designated for gauge attachment, which was selected as the center of the scaffold. This adhesive also filled micro-porosities in the 3D printed scaffold structure to create a stable structure for gauge attachment. Simultaneously, the gauge was then gently removed from the glass slab, and a small amount of adhesive was applied to the backing, after which the gauge was attached to the scaffold surface, ensuring full contact. Gauges were aligned by marking a position on the scaffold that corresponded with the axes of the gauges, as indicated in the rosette (Figure 1). The assemblies were left to cure undisturbed at ambient room temperature (22 °C–24 °C) for a minimum of 12 h (vendor recommendation) to allow complete adhesive polymerization and mechanical fixation of the strain gauges.

**FIGURE 1:**
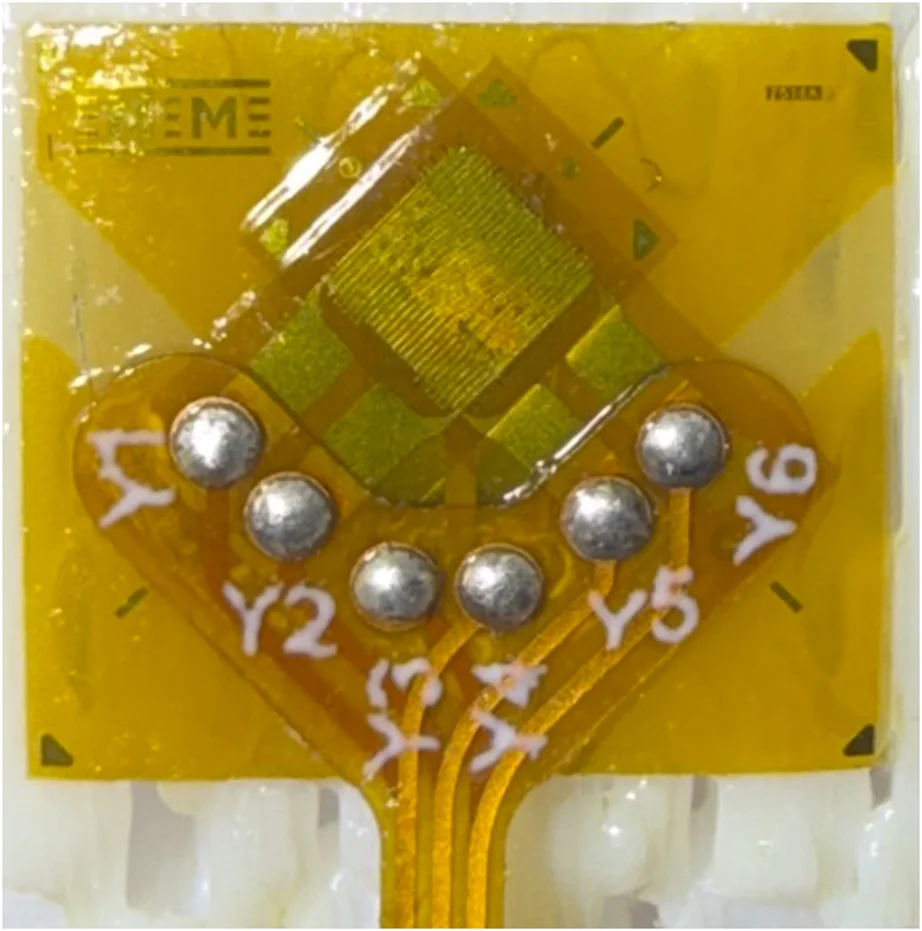
Rosette strain gauges (C4A-29-031WWA-350) from Micro-Measurements.

### 2.2 Sleeve design and fabrication

The endoprosthetic sleeve was fabricated using Facilan™ polycaprolactone (PCL) 100 Filament (3D4Makers, Netherlands), a medical-grade PCL (80 kDa) selected for its biocompatibility, tunable biodegradability, and low-melting-point processability. PCL is FDA approved for numerous orthopedic indications. The sleeve was designed to encapsulate the 3D-printed gyroid scaffold with close tolerance to ensure mechanical load transfer. Scaffold and sleeve dimensions were derived directly from microcomputed tomography (μCT) imagery of the host bone to ensure anatomical congruity and efficient load transfer across the bone–scaffold–sleeve interface. The sleeve was designed to be 1 mm shorter than the scaffold at both ends, to be consistent with an ongoing *in vivo* study.

The design was parametrically modeled in SolidWorks (Dassault Systèmes) 3D CAD to achieve a slip-fit tolerance containing the scaffold. The design included 70.43 percent fenestration, achieved by inserting windows uniformly throughout sleeve walls. The finalized geometries were exported as STL files and processed in PrusaSlicer (Prusa Research, Czech Republic), where layer height and print speed were changed according to material manufacturer specifications. Fabrication was performed using a Prusa MK3 (Prusa Research, Czech Republic) melt-extrusion 3D printer, with nozzle temperature calibrated to 125 °C–135 °C to accommodate the thermal characteristics of PCL. Post-processing was limited to the removal of minor surface artifacts.

### 2.3 Bone preparation

Necropsied ovine hindlimbs were obtained from purely biomechanical studies where no biologic agents or other pharmaceuticals were used. All soft tissues, including muscle, tendon, and periosteum, were meticulously dissected from the surface of the bones using scalpels and periosteal elevators, with special care taken to avoid damaging the underlying cortical structure. The metatarsal bones were carefully isolated from surrounding tarsal and phalangeal structures through dissection. Throughout the dissection, all bone specimens were continuously maintained in chilled PBS to prevent dehydration and degradation.

Following isolation, each metatarsal bone was prepared for a continuous segmental osteotomy to create a 3 cm critical defect in the mid-diaphysis. A 3 cm defect is considered critical in an ovine metatarsus (Sparks et al., 2020; Viateau et al., 2004a). A 3.5 mm Locking Compression Plate (LCP) (Steck et al., 2003) (Movora-Veterinary Orthopedic implants, Augustine, Florida) was positioned axially on the lateral cortex of each bone as it would in this type of surgical intervention. The LCP served both as a guide for drilling and a positional marker for the planned osteotomy. Using the plate as a reference, a 3 cm segment was chosen, ensuring the designated osteotomy region was flanked by three cortical screw holes on both proximal and distal ends. Precision alignment of the plate was confirmed, and the marked region was verified to ensure surgical reproducibility.

Drill guides were secured corresponding to the chosen LCP configuration, and pilot holes were created using a standard battery-powered surgical hand drill. Guides and drill bits were supplied by the manufacturer. Care was taken to drill perpendicularly to the bone surface to preserve axial alignment. Manufacturer-supplied self-tapping cortical screws were then inserted into the predrilled holes using a torque-limited driver to affix the LCP to the bone.

With the LCP still affixed, the central 30 mm section designated for removal was re-measured and confirmed. The plate was temporarily detached, and transverse osteotomies were performed at the marked boundaries using a MicroLux™ Variable Speed Mini Band Saw bone (Berkeley Heights, New Jersey, USA) (Schemitsch, 2017; Viateau et al., 2004a). The excised bone segment was discarded. The LCP was then reassembled on the remaining proximal and distal bone segments, and all screws were reinserted to ensure stable fixation and restoration of alignment. The procedure was performed in accordance with standard surgical protocol for this type of surgical intervention.

Two experimental groups, shown in Figure 2, were examined in this study to evaluate the influence of the polycaprolactone (PCL) sleeve on the strain distribution within the scaffold–bone construct. The first group (NO_SLEEVE, n = 5) consisted of ovine metatarsal bone segments bridged solely by the gyroid scaffold across a critical-sized segmental defect. The second group (SLEEVE, n = 5) incorporated the same configuration with the addition of a PCL sleeve, into which the scaffold was inserted prior to implantation. This configuration provided circumferential confinement of the scaffold and load redistribution by the sleeve. The LCP was used in both groups. In the NO_SLEEVE group, the scaffold was gently press-fit into the defect gap, ensuring full contact with the cut bone surfaces. In the SLEEVE group, the scaffold was first inserted into the PCL sleeve, and the resulting construct was then press-fit into the defect. Tight fit of the scaffold in the sleeve assured effective load transfer from the sleeve into the scaffold. This configuration replicated the mechanical environment of a long-bone segmental defect under fixation, enabling a direct comparison of strain response between the two constructs under identical boundary and loading conditions.

**FIGURE 2:**
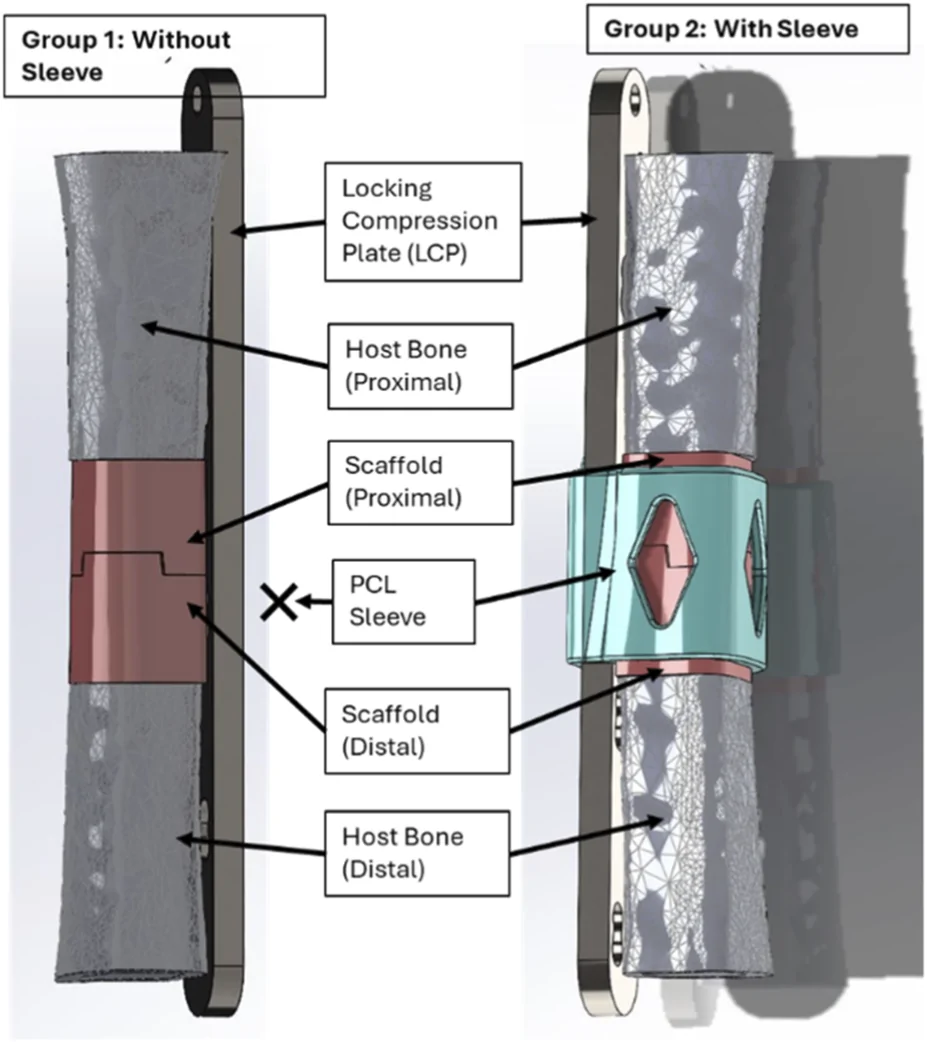
Experimental groups; group 1: NO_SLEEVE and group 2: SLEEVE.

### 2.4 Loading cycle

Mechanical loading was performed using an Incudyne (Cosman, 2003) compression testing machine, configured for sinusoidal cyclic loading. Scaffold–bone assemblies were mounted between custom precision machined stainless steel grips, designed to maintain axial alignment and prevent rotational slip. These grips incorporated set screws to secure the specimens and were adjusted prior to testing so that each compression cycle began under approximately 0 N of preload. The Incudyne applied a sinusoidal load waveform ranging from 0 N to 150 N, corresponding to a loading rate of 1 mm/min, replicating quasi-static physiological compression in ovine walking gait (Rousseau, 2026; ASTM D20 Committee, 2015). This loading cycle is plotted in Figure 3.

**FIGURE 3:**
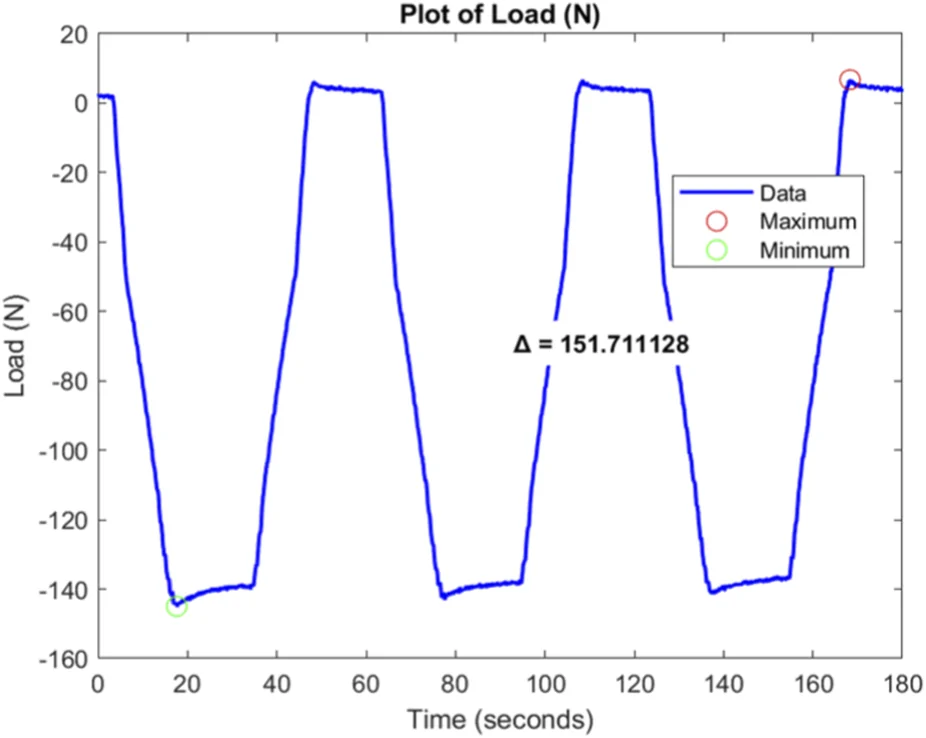
Mechanical loading configured for sinusoidal cyclic loading.

Cyclic compression tests were conducted under identical boundary and loading conditions on all specimens in each experimental group, maintaining all tests within the elastic region. Each test consisted of three sinusoidal loading cycles between 0 N and 150 N, and the full test sequence was repeated three times per specimen to assess repeatability and variability, resulting in nine recorded loading cycles per construct. Incudyne with the testing construct is shown in Figure 4.

**FIGURE 4:**
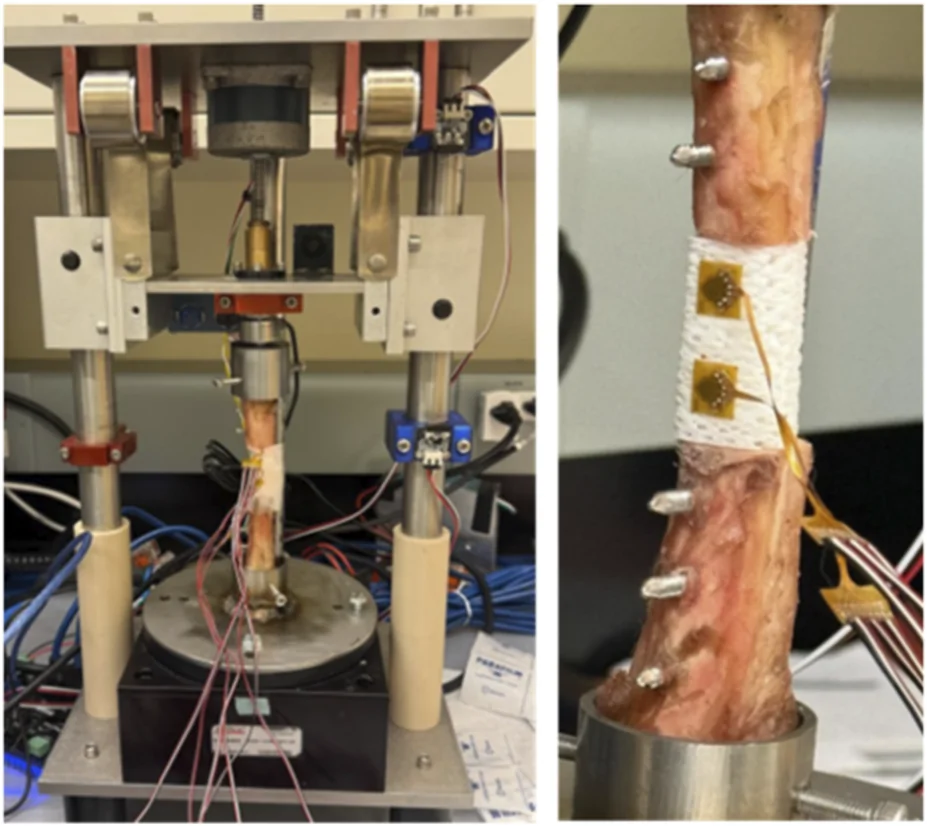
Incudyne compression testing machine with bone-scaffold assembly and strain gauges attached.

To ensure a conservative and representative comparison between groups, the single cycle exhibiting the maximum deviation in peak strain among the three repeated tests for each specimen was selected for reporting. This approach captures the worst-case mechanical response under physiologically relevant peak loading while avoiding artificial averaging that could obscure meaningful differences in strain magnitude between sleeved and unsleeved constructs. The selected peak strain values were then used for subsequent strain transformation, ceramic-equivalent mapping, and statistical analysis.

### 2.5 Strain measurement and transformation

Voltage signals from the strain gauges were recorded in real time using the NI-9219 DAQ module (National Instruments, Texas, USA) interfaced with MATLAB (Natick, Massachusetts, USA). Raw voltage outputs were converted to strain using the manufacturer’s calibration constants. Each cyclic dataset was baseline-corrected to eliminate thermal drift and offset, ensuring that a 0 N load condition corresponded to near-zero strain at the start of every cycle. The differential strain values obtained at peak load (150 N) were collected for analysis. These strain readings were subsequently transformed to principal strains and stresses using the standard strain transformation equations for 0°/±45° rosettes. To evaluate the expected response of an OBM bioceramic scaffold (Durán Hernández et al., 2025) under identical loading, the experimentally measured PLA strains were mapped to OBM-equivalent strains using the ratio of stiffness matrices for PLA and OBM, assuming linear-elastic isotropic behavior for both materials within the applied load range.

During cyclic loading, surface strains were continuously recorded from the rosette gauges mounted on the PLA scaffolds. Raw strain data obtained from the 0°/±45° rosette configuration were resolved into axial (ε_x_), transverse (ε_γ_), and in-plane shear (γ_xγ_) components using standard strain-transformation relations. These components provided a complete description of the local multiaxial strain state under compressive loading.

To relate the measured strain field to material behavior, a material characterization test was first performed on five solid PLA calibration cubes (n = 5) printed using identical parameters (material, orientation, and infill density) as the OBM scaffolds. From the compression tests, the Young’s modulus (E_1_) and Poisson’s ratio (ν_1_) of PLA were determined. These parameters were used to construct the stiffness (C_1_) and compliance (S_1_) matrices defining the constitutive relationship between stress and strain for the PLA scaffold under plane-stress conditions.

Similarly, compression tests were conducted on ceramic OBM cube scaffolds to determine its elastic properties (E_2_, ν_2_), from which the corresponding stiffness (C_2_) and compliance (S_2_) matrices were derived. Assuming that both materials behave linearly and isotropically within the applied load range (≤2% nominal strain), the experimentally measured PLA strain vectors were transformed into ceramic-equivalent strain values using the relationship

$$\varepsilon_{OBM}=S_{OBM}\;\left(C_{PLA}\;\varepsilon_{PLA}\right)$$ (2)

where $S_{OBM}$ and $C_{PLA}$ represent the compliance and stiffness matrices of the OBM and PLA materials, respectively. $\varepsilon_{OBM}\;\&\;\varepsilon_{PLA}$ are the strains in the OBM and PLA scaffolds respectively. This transformation provided an approximation of the strain field that an equivalent ceramic scaffold would experience under the same external loading conditions.

### 2.6 Statistical methods

All strain measurements are reported as median and interquartile range (IQR) given the non-normal distribution of the data. Normality was assessed using the Shapiro-Wilk test which showed that the observed strain distributions exhibited a U-shaped pattern. This is as expected with data concentrated at the extremes rather than the center, violating the assumption of normality. Consequently, between-group comparisons of strain magnitude between the SLEEVE and NO_SLEEVE groups were performed using the Mann-Whitney U test, a non-parametric alternative appropriate for non-normally distributed data. A significance level of α = 0.05 was used for all comparisons, and p-values below this threshold were considered statistically significant.

To assess repeatability across the three repeated loading cycles performed per specimen, a one-way Analysis of Variance (ANOVA) was conducted. No statistically significant differences were found among the three repeated test cycles (p > 0.05), confirming acceptable within-specimen repeatability. For subsequent strain transformation and ceramic-equivalent mapping analyses, maximum deviation in peak strain among the three repeated tests for each specimen was selected to capture the worst-case mechanical response.

Post hoc power analysis was conducted using a two-way ANOVA and the power of the sample size was evaluated. The effect size was quantified using eta square (η2) and the Cohen’s f statistic. All the strain demonstrated very large effect size (η2 = 0.456–0.869; Cohen’s f = 1.15–2.56), exceeding the conventional threshold for a large effect (η2>0.14). The corresponding statistical power for the strain ranged from 0.992 to 1.000, confirming the sample size was more than adequate to reliably detect the observed sleeve induced strain reduction.

All statistical analyses were performed using Microsoft Excel (Microsoft Corporation, Redmond, WA, USA).

## 3 Results

### 3.1 Loading response and data repeatability

Under cyclic loading between 0 and 150 N, the PLA scaffold–bone assemblies exhibited a stable and repeatable load–strain response across successive cycles (Figure 3). The first few cycles showed minor viscoelastic settling, after which strain amplitudes remained consistent, confirming mechanical and adhesive stability of the gauge configuration and the absence of slippage between scaffold and grips. The sinusoidal waveform produced by the Incudyne system yielded uniform stress application throughout the loading path.

Analysis of the strain load response revealed a U-shaped distribution of strain values over each loading cycle, with most strain readings clustered near the extreme load conditions (0 N and 150 N) and comparatively fewer data points near mid-range loads. This behavior is inherent to the sinusoidal loading waveform and reflects the increased dwell time at the peaks and troughs of the cycle. As a result, strain values at maximum load (150 N) exhibited the highest sensitivity to differences in construct configuration and material response.

### 3.2 Strain decomposition and principal directions

Measured voltages from the 0°/±45° rosette gauges were converted to microstrain and decomposed into their orthogonal components: axial strain (ε_x_) along the loading direction, transverse strain (ε_γ_) perpendicular to the axis, and shear strain (γ_xγ_) within the plane of the scaffold face. The dominant deformation mode was axial compression, with smaller but measurable transverse expansion due to Poison’s effects and negligible in-plane shear, confirming that the scaffold walls primarily experienced uniaxial compressive loading. The principal strain orientations determined from Mohr’s circle analysis remained nearly collinear with the loading axis in both test groups, being consistent with the geometric symmetry of the gyroid lattice under axial compression.

### 3.3 Material-property calibration and strain transformation

Compression testing of solid PLA calibration cubes printed under identical parameters yielded an average Young’s modulus (E_1_ ≈ 2.2 GPa) and Poisson’s ratio (ν_1_ ≈ 0.35). These were used to generate the stiffness (C_1_) and compliance (S_1_) matrices governing PLA’s constitutive behavior. Parallel tests on OBM ceramic cubes produced an effective modulus (E_2_ ≈ 6.0 GPa) and Poisson’s ratio (ν_2_ ≈ 0.28), as shown in Figure 5, from which analogous matrices (C_2_, S_2_) were derived.

**FIGURE 5:**
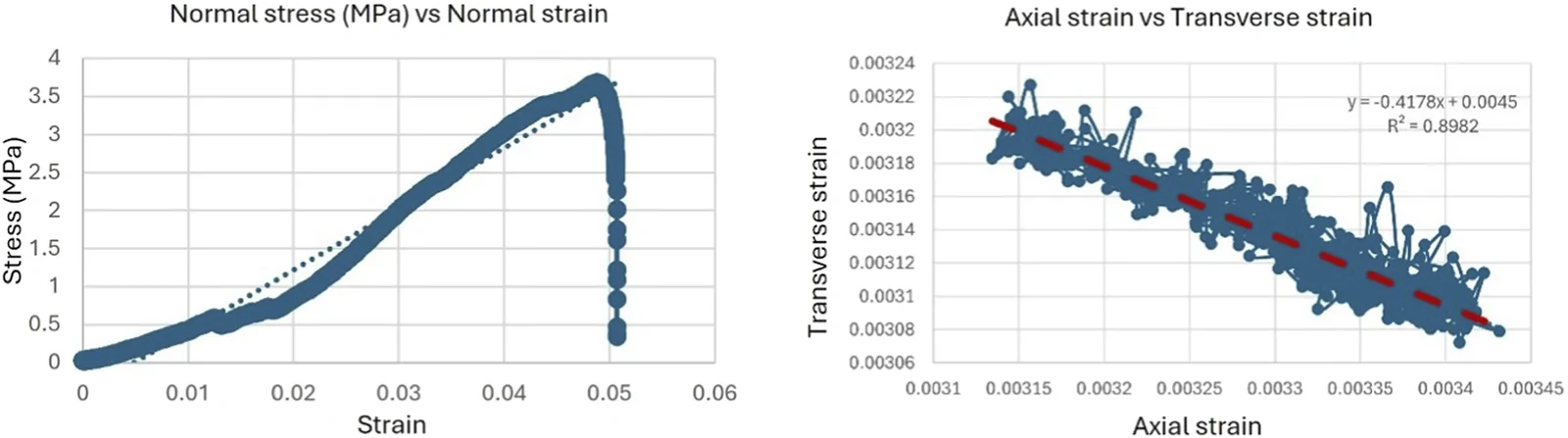
PLA calibration cubes compression testing properties.

Using these properties, the measured PLA strain vectors were converted into equivalent stress states through the PLA stiffness matrix and subsequently mapped through the OBM compliance matrix according to Equation 2, assuming linear-elastic isotropic behavior for both materials within the applied strain range (<2% nominal deformation). This mapping enabled estimation of the strain field that an equivalent ceramic scaffold would experience under identical external loading.

### 3.4 Strain magnitudes and distribution

The transformed OBM-equivalent principal strain magnitudes were found to range between 200 µε and 300 µε, comparable to reported physiological strain levels in healthy cortical bone (100–400 µε) (Cordey and Gautier, 1999). These magnitudes indicate that the scaffolds were loaded within the elastic and mechanobiological window associated with bone maintenance rather than damage or microcracking (Baumer et al., 2024). Across all specimens, axial strain dominated the response, while transverse strain and shear remained an order of magnitude smaller, reflecting efficient load transfer through the gyroid structure.

### 3.5 Effect of the endoprosthetic sleeve

Comparison between the two experimental groups revealed a clear mechanical modulation by the PCL sleeve. Assemblies with the sleeve exhibited a consistent reduction in transverse strain while maintaining similar axial strain amplitudes. On average, the total transverse strain significantly decreased 8.2% in the presence of the PCL sleeve, to ≈ 0.03%.

Cycle-to-cycle variability in recorded strain did not exceed ±5 µε, and the repeatability error between replicate specimens remained within 6% as shown in Figure 6. Baseline noise was below 2 µε following signal filtering, confirming adequate sensitivity of the measurement setup. No evidence of gauge delamination or drift was observed throughout testing, validating the reliability of the recorded strain data for subsequent transformation and comparison.

**FIGURE 6:**
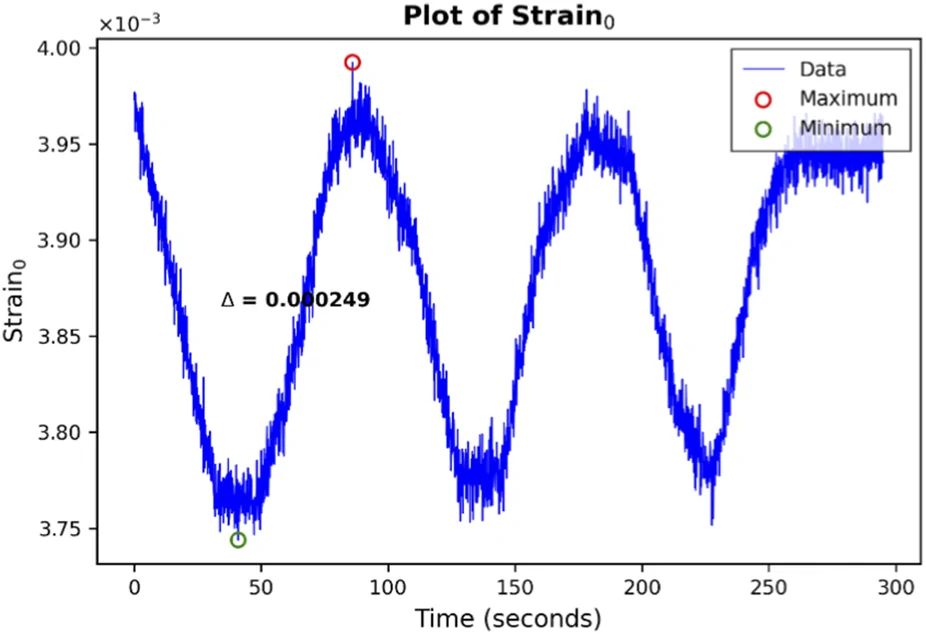
Strain plots and the delta between strain measurements collected.

Figure 7 shows a significant reduction in scaffold transverse strain with the sleeve. The median transverse strain in the SLEEVE group (∼280 µε) was lower than in the NO_SLEEVE group (∼305 µε), with notably tighter interquartile spread and an absence of outliers.

**FIGURE 7:**
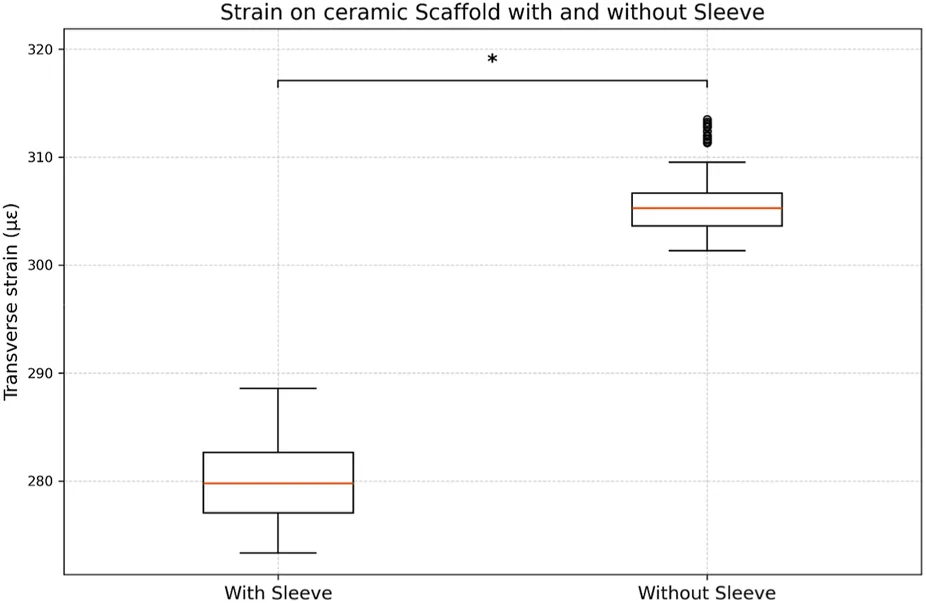
Strain on scaffold surfaces was significantly lower when tested with the sleeve than without it.

## 4 Discussion

The use of PLA as a surrogate scaffold material for strain measurement in bioceramic scaffolds warrants additional justification. Direct instrumentation of OBM comes with inherent challenges in embedding strain gauges directly into the interior of the brittle ceramic scaffold body. The PLA surrogate approach resolves this by decoupling the strain measurement from the ceramic materials, relying instead on mathematical transformation. This approach is analogous to surrogate modelling strategies used in biomechanical testing of bone and implant systems (Álvarez-Blanco et al., 2024), where polymer surrogates have shown to accurately represent mechanical response of harder biological ceramic materials under equivalent loading and boundary conditions. Although the 3D printed PLA scaffolds used in this study are inherently anisotropic due to the layer-by-layer deposition in the melt extrusion process (Cláudio et al., 2022), the unit-cell geometry of the gyroid lattice induces a homogenized mechanical response that is effectively isotropic at the macroscopic scale. This behavior arises because TPMS structures, such as the Gyroid, possess threefold rotational symmetry that constrains their elastic constants to follow cubic symmetry relationships. Finite-element homogenization studies have shown that the Gyroid, Fischer–Koch S, and Diamond TPMS architectures have only three independent elastic constants (C_11_, C_12_, and C_44_), indicating cubic isotropy despite the inherent anisotropy of the printed material (Lu et al., 2019). Consequently, when these unit cells are periodically repeated, the resulting scaffold exhibits nearly uniform mechanical behavior in all principal directions, allowing the use of isotropic elastic approximations for the effective modulus and Poisson’s ratio. Isotropic equivalence described above justifies the transformation of experimentally measured PLA strains to the equivalent ceramic (OBM) response under the same stress state, as the gyroid topology governs the overall elastic symmetry rather than the printing direction of the polymer. Because our applied deformations are small (≲2% nominal strain), both PLA and the OBM ceramic operate in the linear-elastic regime. Therefore, it is reasonable to translate the measured PLA strain state to the ceramic via their constitutive stiffness and compliance matrices.

In determining the 0–150 N cyclic load range for the scaffold-bone assembly, we considered physiological loading of the ovine metatarsal during walking. Kinetic gait analyses in sheep demonstrate that during the stance phase of walking, each limb bears a substantial fraction of the animal’s body weight. For example, in one study the mean peak vertical force (PVF) in the forelimbs was ∼52.5% of body weight, and in the hind limbs ∼38.5% of body weight (stance time ∼66% and ∼69% of the gait cycle respectively) for adult sheep walking at ∼1.06 m/s (Kim and Breur, 2008). Thus, for a ∼60 kg sheep, a single limb could plausibly transmit ∼0.4 × 60 kg × 9.81 m/s^2^ ≈ 235 N during peak load. Given that not all that load is carried exclusively by the scaffold (some load transfers via soft tissue and plate fixation), selecting 150 N loads places our cyclic regime conservatively below the physiological maximum while still capturing relevant mechanobiological strain levels. In effect, this sub-peak loading ensures that the scaffold remains within its elastic regime (as assumed) while subjecting it to a representative fraction of *in vivo* loading, thus realistically modeling the mechanical environment of the defect without risking immediate material damage. It is acknowledged that 150 N does not capture the full spectrum of mechanical demands encountered during unrestricted activity. Ground reaction forces increase substantially with activity intensity, reaching 2-3 times body weight during running, which could translate to a much greater load than the current modeled situation (Kim and Breur, 2008).

The reduction in transverse expansion in the SLEEVE group indicates that the sleeve redistributed load more uniformly across the scaffold perimeter, suppressing local bending of gyroid walls and promoting a more homogeneous compressive stress field. These observations suggest that the compliant PCL sleeve might be acting as an external constraint that selectively attenuates transverse strain while preserving axial compliance as evidenced by the statistically non-significant difference in axial strain between the SLEEVE and NO_SLEEVE groups (p > 0.05). This mechanical behavior is particularly advantageous from a mechanobiological standpoint as lower transverse strain reduces the risk of brittle fracture at gyroid wall junctions, while maintaining axial compliance ensuring that the scaffold continues to transmit physiologically relevant compressive deformation to the surrounding bone tissue, preserving the mechanobiological stimulus considered necessary for bone formation and remodeling (Claes et al., 1998).

The reduction in transverse strain in the SLEEVE group, with notably tighter interquartile spread with an absence of outliers, suggests that the sleeve not only reduces mean strain magnitude but also dampens peak strain events that could initiate crack propagation in brittle ceramic scaffolds. The NO_SLEEVE group exhibited several high-strain outliers exceeding 310 µε, which may represent transient loading events particularly hazardous to ceramic constructs. These findings support a hypothesis that the externalized PCL sleeve functions as a mechanical buffer, redistributing axial compressive loads away from the scaffold body and reducing both the magnitude and variability of strain experienced under cyclic physiological loading.

The strain-modulation action of the sleeve may contribute to one of the central mechanobiological barriers to successful critical-sized defect repair: finding the appropriate balance between a mechanical environment that is stable enough to promote rapid ossification while avoiding the detrimental effects of excessive rigidity, motion, or strain. In the present study, the endoprosthetic sleeve was designed to complement an LCP by providing controlled immobilization and support for highly porous scaffolds that might otherwise be too fragile to survive under human-scale loading conditions. This is an important advancement because conventional LCP fixation can be overly stiff, creating a non-ideal strain environment that may suppress bone formation in the defect region, whereas insufficient fixation permits excessive interfragmentary movement, prolongs the chondral phase, increases fibrous tissue formation, and reduces callus strength and stiffness. By reducing strain within the scaffold, the sleeve could potentially shift the repair environment toward the mechanobiologically favorable range (Claes et al., 1998; Claes and Heigele, 1999). Thus, this approach may hint at clinical potential of the sleeve as a strategy to stabilize large defects and optimize the balance between fixation rigidity and biomechanically favorable micromotion needed for robust bone regeneration.

No prior study has evaluated OBM scaffolds with gyroid architecture *in vivo* (our group is the first to fabricate them). Therefore, any suggestion of their osteogenic potential would be hypothetical. However, numerous deproteinized bone mineral grafting materials like OBM have been well studied and have demonstrated clinical success in numerous commercial products (e.g., Bio-OSS®). These biomaterials are among the most effective bone grafting materials for promoting osteogenesis relative to alternate substitutes (Dhanavelu et al., 2025), consistently demonstrating enhanced bone formation and graft integration in multiple animal models (Abdelmoneim et al., 2024). Moreover, gyroid TPMS scaffold architectures have demonstrated strong osteogenic and angiogenic performance *in vivo* across multiple material systems (Maevskaia et al., 2024; Xian et al., 2025). Taken together, these two factors provide a strong mechanistic basis for anticipating effective bone regeneration from OBM gyroid scaffolds, a hypothesis that is currently being evaluated by our group in an *in vivo* ovine study. The strain modulation demonstrated by the endoprosthetic sleeve in the present study therefore has potential clinical relevance: by reducing scaffold strains within a range considered beneficial for new bone formation (Claes and Heigele, 1999), the sleeve may create more favorable conditions that may enhance the osteogenic potential of these fragile bioceramic scaffolds.

All bone specimens were stored frozen when not in use and thawed to room temperature for at least 2 hours prior to testing to maintain hydration and prevent microstructural degradation. Previous work has shown that freezing at −20 °C has negligible effects on cortical bone mechanics, with any changes in indentation or stiffness parameters remaining within natural sample variability and well below 15% (Kaye, 2012). Therefore, the freezing–thawing process used in this study is unlikely to have influenced the measured strain response.

A notable limitation of this study is that strain measurements were confined to the scaffold outer surface, not including the bone–scaffold interfaces at the proximal and distal ends or the inter-segmental interface. Body-level strain measurements provide valuable insight into the global mechanical environment within the scaffold. However, they do not capture interfacial strain gradients that may be associated with small, localized defects in scaffold architecture, which can strongly influence osseointegration, load transfer, and failure initiation, particularly in large bone defects. Furthermore, localized strain amplification at the scaffold–bone interface may play a dominant role in bone formation or fibrous tissue development, particularly in constructs stabilized with locking compression plates, therefore surface strain measurement may actually underestimate peak local strains at these regions. Moreover, the use of surface-mounted rosette strain gauges limited the measurements to localized, planar strain states, precluding resolution of three-dimensional strain heterogeneity, bending-dominated regions, and through-thickness gradients inherent to gyroid architectures. Although adequate for comparative evaluation of sleeved and unsleeved constructs, this method cannot fully characterize internal deformation mechanisms. Future work should incorporate volumetric strain measurement techniques, such as tomographic digital volume correlation (DVC) (Madi et al., 2013) or embedded fiber-optic strain sensors, in conjunction with multiaxial and cyclic fatigue loading to more accurately replicate *in vivo* conditions and enhance the physiological relevance and predictive capability of the experimental framework.

Future work might also include finite element analysis (FEA). The primary reason FEA was not pursued in this work is the significant geometric and computational complexity inherent to gyroid scaffold architecture. Achieving a well resolved, high-quality tetrahedral mesh over a 3cm scaffold at 70% porosity and 1mm pore size requires either specialized implicit geometry meshing tools or voxel-based meshing, neither of which was available within the scope and timeline of this study. The primary objective of this study was to demonstrate the strain-modulating capability of the sleeve; therefore, assessment of the detailed 3D strain domain within the scaffold was beyond its scope.

Only short-term cyclic loading was evaluated in this study. Although long-term fatigue behavior under repeated physiological loading warrants consideration, the risk of fatigue-related damage is substantially reduced by the elastic loading regime employed. Applied loads were conservatively maintained below physiological peak levels while still generating mechanobiologically relevant strain magnitudes. Consequently, the scaffold remained within its elastic deformation regime while experiencing a representative fraction of *in vivo* loading, providing a realistic approximation of the mechanical environment within the defect. The primary objective of this study was to demonstrate the strain-modulating capability of the sleeve; therefore, assessment of long-term degradation mechanisms was beyond its scope.

Nevertheless, additional time-dependent mechanical phenomena may influence long-term performance. For example, polycaprolactone (PCL) may undergo gradual viscoelastic creep under sustained loading, potentially resulting in a progressive reduction in sleeve stiffness. Similarly, highly porous brittle ceramics can accumulate subcritical microcracks over time, even when loaded within their nominal elastic regime. Importantly, the strain-reduction effect provided by the sleeve is expected to be beneficial in both cases, as lower strain magnitudes reduce the driving forces associated with creep-induced compliance changes and subcritical crack growth. However, these long-term mechanisms remain important topics for future investigation.

An important consideration for any polymer-based reinforcement strategy is its long-term degradation behavior under physiological conditions. The sleeve evaluated in this study was fabricated from medical-grade 80 kDa polycaprolactone (PCL), a semicrystalline polyester that degrades primarily through bulk hydrolysis of its aliphatic ester bonds. PCL exhibits relatively slow hydrolytic degradation kinetics, with reports indicating an approximately 80%–90% reduction in number-average molecular weight over 2–3 years *in vivo*. Previous studies of three-dimensional PCL scaffolds have demonstrated minimal degradation during the early implantation period, with negligible changes in molecular weight after 6 months and mass losses of only approximately 7% in PCL composite scaffolds. These findings suggest that the material largely retains its structural integrity during a substantial portion of the bone healing process.

The slow degradation profile of PCL is advantageous for the present application because the sleeve is intended to provide mechanical reinforcement during the critical early phases of bone regeneration, which typically extend from 6 to 18 months in critical-sized defects. As new bone forms and remodels, load-bearing responsibility is progressively transferred from the implant to the regenerating tissue. Based on its reported degradation characteristics, the 80 kDa PCL used in this study is expected to maintain mechanical integrity for approximately 18–24 months, thereby spanning the period of bone formation and remodeling before undergoing gradual resorption (Lam et al., 2009).

## 5 Conclusion

This study demonstrated the ability of an endoprosthetic sleeve to significantly reduce scaffold surface strain under ovine walking loads. Sleeves were designed to be easy to install in common segmental, extra-articular, large bone defects in conjunction with a locking compression plate. This approach enables the use of engineered scaffolds composed of promising biomaterials that might otherwise be too brittle for use in load bearing indications. It is important to clarify that this study does not intend to provide a basis to claim that the OBM scaffold material improves osteogenesis. *In vivo* or *in vitro* performance has not been evaluated in this study. Rather, the externalized sleeve provides mechanical reinforcement and transverse strain modulation without altering the scaffold material or internal architecture, maintaining the osteoconductive capacity known to be associated with bioceramic scaffolds, while extending their viable loading range for large bone defect remediation. This alternative reinforcement strategy potentially reopens opportunities for further exploration of promising biomaterials that were rejected due to inadequate mechanical properties. Moreover, this strategy can be extended to other impactful indications such as oral-maxillofacial reconstruction.

## Data Availability

The raw data supporting the conclusions of this article will be made available by the authors, without undue reservation.

## References

[B1] Abdelmoneim D. Coates D. E. Schmidlin P. Botter S. Li K. C. Porter G. C. (2024). *In vivo* healing of low temperature deproteinized bovine bone xenograft in a rabbit cranial model. J. Biomed. Mater. Res. 112 (9), 1436–1450. 10.1002/jbm.a.37693 38466022

[B2] Álvarez-Blanco M. Infante-García D. Marco M. Giner E. Miguélez M. H. (2024). Development of bone surrogates by material extrusion-based additive manufacturing to mimic flexural mechanical behaviour and fracture prediction *via* phase-field approach. Comput. Methods Programs Biomed. 254, 108287. 10.1016/j.cmpb.2024.108287 38908222

[B3] ASTM D20 Committee (2015). D695-15, Standard Test Method for Compressive Properties of Rigid Plastics. West Conshohocken, PA: ASTM International. 10.1520/D0695-15

[B4] Baumer V. Isaacson N. Kanakamedala S. McGee D. Kaze I. Prawel D. (2024). Comparing ceramic Fischer-Koch-S and gyroid TPMS scaffolds for potential in bone tissue engineering. Front. Bioeng. Biotechnol. 12, 1410837. 10.3389/fbioe.2024.1410837 39193226 PMC11347304

[B5] Campana V. Milano G. Pagano E. Barba M. Cicione C. Salonna G. (2014). Bone substitutes in orthopaedic surgery: from basic science to clinical practice. J. Mater Sci. Mater Med. 25 (10), 2445–2461. 10.1007/s10856-014-5240-2 24865980 PMC4169585

[B6] Cao C. Huang P. Prasopthum A. Parsons A. J. Ai F. Yang J. (2022). Characterisation of bone regeneration in 3D printed ductile PCL/PEG/hydroxyapatite scaffolds with high ceramic microparticle concentrations. Biomater. Sci. 10 (1), 138–152. 10.1039/D1BM01645H 34806738

[B7] Castro A. P. G. Pires T. Santos J. E. Gouveia B. P. Fernandes P. R. (2019). Permeability *versus* design in TPMS scaffolds. Materials 12 (8), 1313. 10.3390/ma12081313 31013656 PMC6515433

[B8] Claes L. E. Heigele C. A. (1999). Magnitudes of local stress and strain along bony surfaces predict the course and type of fracture healing. J. Biomechanics 32 (3), 255–266. 10.1016/S0021-9290(98)00153-5 10093025

[B9] Claes L. E. Heigele C. A. Neidlinger-Wilke C. Kaspar D. Seidl W. Margevicius K. J. (1998). Effects of mechanical factors on the fracture healing process. Clin. Orthop. Relat. Res. 355S, S132–S147. 10.1097/00003086-199810001-00015 9917634

[B10] Clark J. N. Tavana S. Heyraud A. Tallia F. Jones J. R. Hansen U. (2020). Quantifying 3D strain in scaffold implants for regenerative medicine. Materials 13 (17), 3890. 10.3390/ma13173890 32899192 PMC7504351

[B11] Cláudio R. A. Dupont J. Baptista R. Leite M. Reis L. (2022). Behaviour evaluation of 3D printed polylactic acid under compression. J. Mater. Res. Technol. 21, 4052–4066. 10.1016/j.jmrt.2022.10.042

[B12] Cordey J. Gautier E. (1999). Strain gauges used in the mechanical testing of bones part II: *‘in vitro’* and *‘in vivo’* technique. Injury 30, SA14–SA20. 10.1016/S0020-1383(99)00121-7 10645364

[B13] Cosman C. M. (2003). “Characterization of shear stiffness and cell metabolism in chondrocyte-seeded self-assembling peptide hydrogel scaffolds,”. Cambridge, MA, Jun: Massachusetts Institute of Technology.

[B14] Dhanavelu P. Nandagopal K. G. Ganesh N. S. Athira K. S. Quadri A. Francis M. (2025). *In vitro* evaluation of the osteogenic potential of different bone graft materials in oral surgery. J. Pharm. Bioallied Sci. 17 (Suppl. 3), S2656–S2658. 10.4103/jpbs.jpbs_820_25 41164708 PMC12563632

[B15] Durán Hernández O. Baumer V. Marrero G. Karumanchi S. Prawel D. (2025). Fabrication and characterization of highly porous gyroid scaffolds composed of deproteinized bone mineral. JFB 16 (4), 119. 10.3390/jfb16040119 40278227 PMC12028333

[B16] Epari D. R. Schell H. Bail H. J. Duda G. N. (2006). Instability prolongs the chondral phase during bone healing in sheep. Bone 38 (6), 864–870. 10.1016/j.bone.2005.10.023 16359937

[B17] Green E. Lubahn J. D. Evans J. (2005). Risk factors, treatment, and outcomes associated with nonunion of the midshaft humerus fracture. J. Surg. Orthop. Adv. 14 (2), 64–72. 16115430

[B18] Kaye B. (2012). The effects of freezing on the mechanical properties of bone. TOBONEJ 4 (1), 14–19. 10.2174/1876525401204010014

[B19] Kim J. Breur G. J. (2008). Temporospatial and kinetic characteristics of sheep walking on a pressure sensing walkway. Can. J. Veterinary Res. 72, 50–55. 18214162 PMC2117367

[B20] Komurlu E. Cihangir F. Kesimal A. Demir S. (2016). Effect of adhesive type on the measurement of modulus of elasticity using electrical resistance strain gauges. Arab. J. Sci. Eng. 41 (2), 433–441. 10.1007/s13369-015-1837-0

[B21] Kutrolli M. Pereira N. S. Ghanbariamin D. Arnold K. M. Deymier A. Samandari M. (2026). Three-dimensional printing of polycaprolactone scaffolds with multiscale porosity for bone tissue engineering. ACS Biomater. Sci. Eng. 12, 4894–4906. 10.1021/acsbiomaterials.6c00276 42619584

[B22] Lam C. X. F. Hutmacher D. W. Schantz J. Woodruff M. A. Teoh S. H. (2009). Evaluation of polycaprolactone scaffold degradation for 6 months *in vitro* and *in vivo* . J. Biomed. Mater. Res. 90A (3), 906–919. 10.1002/jbm.a.32052 18646204

[B23] Liang H.-Y. Lee W. K. Hsu J. T. Shih J. Y. Ma T. L. Vo T. T. T. (2024). Polycaprolactone in bone tissue engineering: a comprehensive review of innovations in scaffold fabrication and surface modifications. J. Funct. Biomater. 15 (9), 243. 10.3390/jfb15090243 39330219 PMC11433047

[B24] Lu Y. Zhao W. Cui Z. Zhu H. Wu C. (2019). The anisotropic elastic behavior of the widely-used triply-periodic minimal surface based scaffolds. J. Mech. Behav. Biomed. Mater. 99, 56–65. 10.1016/j.jmbbm.2019.07.012 31344523

[B25] Madi K. Tozzi G. Zhang Q. H. Tong J. Cossey A. Au A. (2013). Computation of full-field displacements in a scaffold implant using digital volume correlation and finite element analysis. Med. Eng. Phys. 35 (9), 1298–1312. 10.1016/j.medengphy.2013.02.001 23473631

[B26] Maevskaia E. Ghayor C. Bhattacharya I. Guerrero J. Weber F. E. (2024). TPMS microarchitectures for vertical bone augmentation and osteoconduction: an *in vivo* study. Materials 17 (11), 2533. 10.3390/ma17112533 38893806 PMC11173251

[B27] Marsell R. Einhorn T. A. (2011). The biology of fracture healing. Injury 42 (6), 551–555. 10.1016/j.injury.2011.03.031 21489527 PMC3105171

[B28] Melchels F. P. W. Bertoldi K. Gabbrielli R. Velders A. H. Feijen J. Grijpma D. W. (2010). Mathematically defined tissue engineering scaffold architectures prepared by stereolithography. Biomaterials 31 (27), 6909–6916. 10.1016/j.biomaterials.2010.05.068 20579724

[B29] Ng V. Y. (2012). Risk of disease transmission with bone allograft. Orthopedics 35 (8), 679–681. 10.3928/01477447-20120725-04 22868589

[B30] Pilia M. Guda T. Appleford M. (2013). Development of composite scaffolds for load-bearing segmental bone defects. BioMed Res. Int. 2013, 1–15. 10.1155/2013/458253 23984363 PMC3745947

[B31] Pobloth A.-M. Checa S. Razi H. Petersen A. Weaver J. C. Schmidt-Bleek K. (2018). Mechanobiologically optimized 3D titanium-mesh scaffolds enhance bone regeneration in critical segmental defects in sheep. Sci. Transl. Med. 10 (423), eaam8828. 10.1126/scitranslmed.aam8828 29321260

[B32] Rousseau C. (2026). Interlaboratory Study to Establish Precision Statements for ASTM D6641-14, Standard Test Method for Compressive Properties of Polymer Matrix Composite Materials Using a Combined Loading Compression (CLC) Test Fixture.

[B33] Schell H. Epari D. R. Kassi J. P. Bragulla H. Bail H. J. Duda G. N. (2005). The course of bone healing is influenced by the initial shear fixation stability. J. Orthop. Res. 23 (5), 1022–1028. 10.1016/j.orthres.2005.03.005 15878254

[B34] Schemitsch E. H. (2017). Size matters: defining critical in bone defect size. J. Orthop. Trauma 31 (5), S20–S22. 10.1097/BOT.0000000000000978 28938386

[B35] Schmidt A. H. (2021). Autologous bone graft: is it still the gold standard? Injury 52, S18–S22. 10.1016/j.injury.2021.01.043 33563416

[B36] Sparks D. S. Saifzadeh S. Savi F. M. Dlaska C. E. Berner A. Henkel J. (2020). A preclinical large-animal model for the assessment of critical-size load-bearing bone defect reconstruction. Nat. Protoc. 15 (3), 877–924. 10.1038/s41596-019-0271-2 32060491

[B37] Steck R. Gatzka C. Schneider E. Niederer P. Tate M. L. (2003). Measurement of bone surface strains on the sheep metacarpus *in vivo* and *ex vivo* . Vet. Comp. Orthop. Traumatol. 16 (01), 38–43. 10.1055/s-0038-1632754

[B38] Viateau V. Guillemin G. Yang Y. C. Bensaid W. Reviron T. Oudina K. (2004). A technique for creating critical-size defects in the metatarsus of sheep for use in investigation of healing of long-bone defects. Ajvr 65 (12), 1653–1657. 10.2460/ajvr.2004.65.1653 15631029

[B39] Xian G. Charbonnier B. Bensidhoum M. Potier E. Margottin M. Sheng P. (2025). Gyroid‐structured scaffolds guide uniform ossification and modulate vascular morphology during rat calvarial bone defect regeneration. J. Biomed. Mater. Res. 113 (9), e37978. 10.1002/jbm.a.37978 40884424

[B40] Yang Y. P. Labus K. M. Gadomski B. C. Bruyas A. Easley J. Nelson B. (2021). Osteoinductive 3D printed scaffold healed 5 Cm segmental bone defects in the ovine metatarsus. Sci. Rep. 11 (1), 6704. 10.1038/s41598-021-86210-5 33758338 PMC7987996

[B41] Younger E. M. Chapman M. W. (2026). Morbidity at Bone Graft Donor Sites. 10.1097/00005131-198909000-00002 2809818

